# Global Taxonomic Diversity of Living Reptiles

**DOI:** 10.1371/journal.pone.0059741

**Published:** 2013-03-27

**Authors:** Daniel Pincheira-Donoso, Aaron M. Bauer, Shai Meiri, Peter Uetz

**Affiliations:** 1 Laboratory of Evolutionary Ecology of Adaptations, School of Life Sciences, University of Lincoln, Riseholme Park, Lincoln, Lincolnshire, United Kingdom; 2 Department of Biology, Villanova University, Villanova, Pennsylvania, United States of America; 3 Department of Zoology, Tel Aviv University, Tel Aviv, Israel; 4 Center for the Study of Biological Complexity, Virginia Commonwealth University, Richmond, Virginia, United States of America; Consiglio Nazionale delle Ricerche (CNR), Italy

## Abstract

Reptiles are one of the most ecologically and evolutionarily remarkable groups of living organisms, having successfully colonized most of the planet, including the oceans and some of the harshest and more environmentally unstable ecosystems on earth. Here, based on a complete dataset of all the world’s diversity of living reptiles, we analyse lineage taxonomic richness both within and among clades, at different levels of the phylogenetic hierarchy. We also analyse the historical tendencies in the descriptions of new reptile species from Linnaeus to March 2012. Although (non-avian) reptiles are the second most species-rich group of amniotes after birds, most of their diversity (96.3%) is concentrated in squamates (59% lizards, 35% snakes, and 2% amphisbaenians). In strong contrast, turtles (3.4%), crocodilians (0.3%), and tuataras (0.01%) are far less diverse. In terms of species discoveries, most turtles and crocodilians were described early, while descriptions of lizards, snakes and amphisbaenians are multimodal with respect to time. Lizard descriptions, in particular, have reached unprecedented levels during the last decade. Finally, despite such remarkably asymmetric distributions of reptile taxonomic diversity among groups, we found that the distributions of lineage richness are consistently right-skewed, with most clades (monophyletic families and genera) containing few lineages (monophyletic genera and species, respectively), while only a few have radiated greatly (notably the families Colubridae and Scincidae, and the lizard genera *Anolis* and *Liolaemus*). Therefore, such consistency in the frequency distribution of richness among clades and among phylogenetic levels suggests that the nature of reptile biodiversity is fundamentally fractal (i.e., it is scale invariant). We then compared current reptile diversity with the global reptile diversity and taxonomy known in 1980. Despite substantial differences in the taxonomies (relative to 2012), the patterns of lineage richness remain qualitatively identical, hence reinforcing our conclusions about the fractal nature of reptile biodiversity.

## Introduction

Reptiles are among the most remarkable components of global biodiversity. The ecological and evolutionary role of these organisms has played a primary part in the origin and subsequent radiations of amniote vertebrates, and in the function of modern-day ecosystems [1]–[3]. Evolutionary milestones in reptiles past, such as the acquisition of water-independent reproduction that resulted in their establishment as the first fully-terrestrial vertebrates, and their universally known Mesozoic proliferation followed by mass extinctions (most notably embodied by dinosaurs, ichthyosaurs and pterosaurs), are among the most important events in vertebrate evolutionary history [3], [4]. Likewise, as major components of current biotas globally, reptiles have successfully invaded most areas of the world, except the poles, and including the oceans [3], [5]. As a result of radiations over hundreds of millions of years, reptiles have accumulated a vast diversity of morphological, behavioural, ecological, life history, and defensive strategies to cope with the selective demands they have encountered [3], [6]–[10]. These and other features have earned reptiles a central role as model systems for evolutionary and ecological research [4], [11].

The evolutionary history of reptiles has given rise to considerably asymmetric species-richness among phylogenetic groups. While turtles, crocodilians and tuataras (non-squamate reptiles) combined do not reach 350 species (and are, in turn, considerably asymmetric among themselves), the clade Squamata (lizards, snakes and amphisbaenians) has diversified into more than 9,100 species [12], [13]. These patterns of species richness are, to some extent, mirrored by order-level geographic range sizes, as both turtles and crocodilians despite being widespread around the world, have failed to radiate in cold climates, where some squamate lineages, in contrast, have successfully proliferated [3], [4], [12]. As a result, squamates have consolidated as the most successful lineage among living reptiles in terms of species richness, morphological and ecological diversity, and as one of the most successful orders among terrestrial vertebrates in general. Indeed, some of the most remarkable examples of vertebrate evolutionary radiations have occurred within squamates. Particularly notorious cases are the hyperdiverse iguanian genera *Anolis*, within which nearly 400 species are known from tropical America [11], [13], and *Liolaemus*, consisting of 220+ species occurring across one of the widest climatic and ecological ranges known among living reptiles [14], [15]. These two lizard genera are the most species-rich among amniote vertebrates on earth. It is worth noting, however, that several authors [16], [17] have suggested splitting of *Anolis* into multiple genera.

These asymmetries in taxonomic richness among reptile clades reflect major differences in the evolutionary dynamics that underlie the way lineages radiate and go extinct [18], [19]. For example, the antagonistic effects of evolvability (the capacity of organisms to adapt to changing environments) and genetic constraints (tendency for phylogenetic niche conservatism) on the potential of clades to radiate and proliferate, or the roles that key innovations play in the tempo of lineage diversification [20]–[23] and extinction [24]. For these reasons, an understanding of the phylogenetic distribution of species richness within major groups of organisms can have, in turn, profound implications for understanding the way biodiversity evolves [25]. However, studies aimed to explore patterns of taxonomic diversity among entire lineages (e.g., reptiles) must meet the challenge of having a comprehensive account of the species known within each clade. Multiple attempts have been made to assemble global datasets of amphibians, birds and mammals, from which a number of patterns of diversity have been shown (e.g., [26]–[28]). In contrast, such global-scale analyses are almost entirely lacking for reptiles. Some studies, for instance, have concentrated on particular groups, e.g., lizards [29] or turtles [30]. For reptiles in general, only a brief account of their patterns of species richness was presented more than a decade ago by Uetz [12]. However, by then the total number of known species was considerably lower than it is today (∼80% of current diversity; see results and [31]), and phylogenetic relationships among and within major lineages were poorly resolved and based on much more restricted datasets than currently available. In a more recent study, Ricklefs et al. [25] investigated the phylogenetic patterns of diversity among 36 clades (at subfamily level) of squamate reptiles. These authors revealed a general tendency for exceptionally rich clades to be rare, while smaller clades to be the norm. More generally, previous studies have suggested that the structural organization of biodiversity at different taxonomic levels is fundamentally fractal (i.e., scale invariant) [32]. However, whether this pattern of diversity distribution is consistent among reptiles in general, and among phylogenetic levels of taxonomic hierarchy, i.e., whether this pattern of diversity is fractal, remains unknown.

Here, we investigate the patterns of reptile lineage taxonomic diversity both within and among clades, at different levels of the phylogenetic hierarchy, based on a comprehensive dataset of all living reptile species described and considered valid until March 2012. These data are currently compiled in the Reptile Database [13]. In addition, we complement these analyses with an examination of the historical rates of reptile species descriptions in the scientific literature from Linnaeus [33] to 2012.

## Results

### Patterns of Species Descriptions

**Figure 1:**
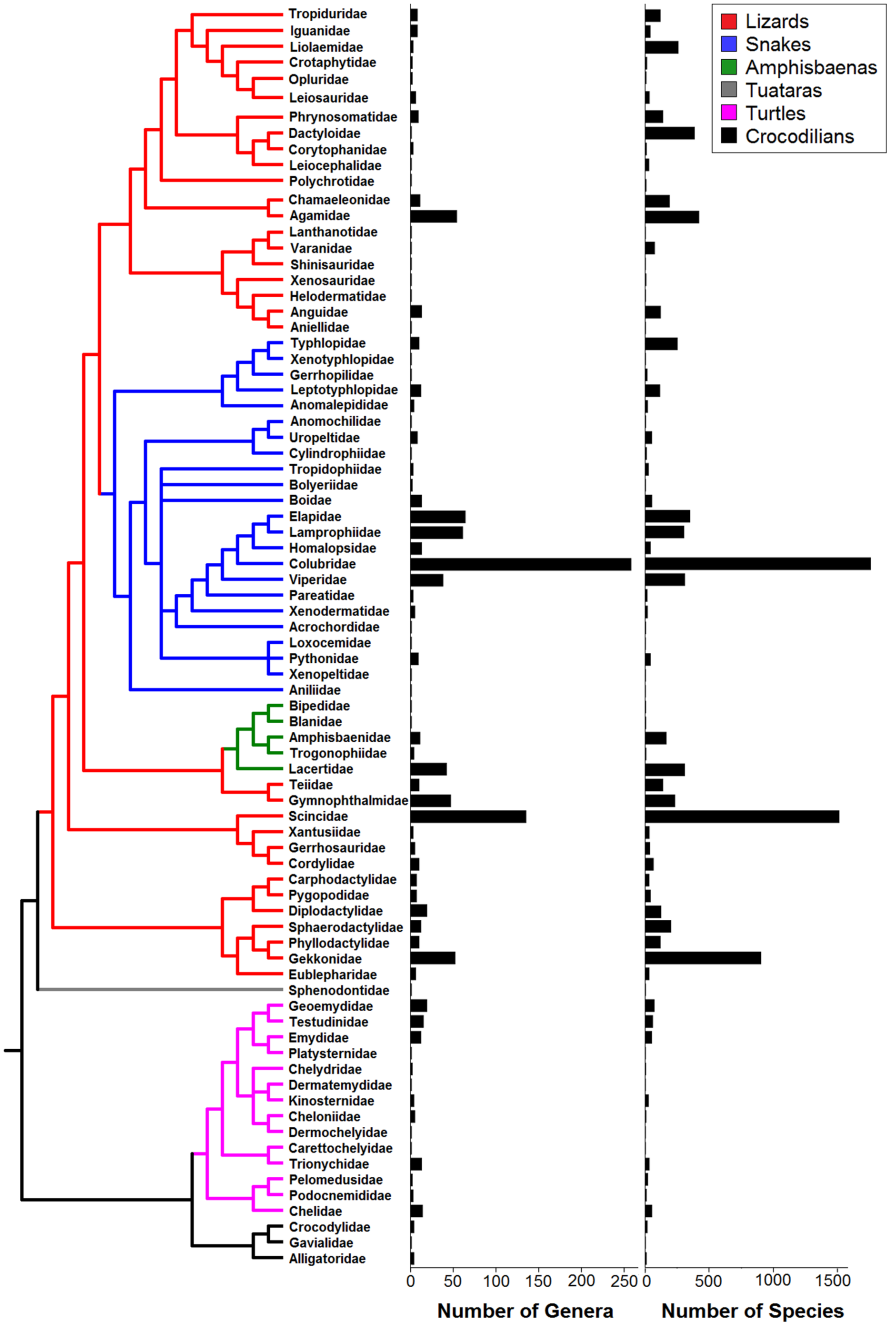
Phylogenetic distribution of genera and species diversity among currently known families of living reptiles. The six major reptile groups are differentiated in colours, as detailed in the top-right box. The lizard families Dibamidae and Hopolcercidae, and the amphisbaenian families Cadeidae and Rhineuridae are not shown because of conflicting phylogenetic information. Birds and other vertebrates have been excluded from the tree.

The world’s known diversity of living reptiles has reached 9,546 species at the time of this analyses (March 2012), of which 25 (0.3%) are crocodilians, 327 (3.4%) are turtles, and one (0.01%) is the tuatara [34]. The remaining 9,193 (96.3%) species are squamates (lizards, snakes and amphisbaenians) (Table 1; Fig. 1). Within squamates, most diversity is concentrated in the paraphyletic suborder Sauria (lizards – 5,634 species) and in the monophyletic suborder Serpentes (snakes – 3,378 species), whereas only 181 species are amphisbaenians (suborder Amphisbaenia). Compared with the account presented 12 years ago by Uetz [12], these species counts represent increases of 32 species (10.8% increase) of turtles, and two crocodilians (8.7% increase) [35], [36], whereas the taxonomic richness of tuataras has declined from two to one as a result of recent genetic evidence [34]. Among squamates, a remarkable 1,647 (21.8% increase) species were added during this period, of which 1,164 species are lizards (26% increase), 458 species are snakes (15.7% increase), and 25 species are amphisbaenians (16% increase). Collectively, thus, the entire known diversity of living reptiles (based on species descriptions considered valid) has increased by 1,680 species (21.4% increase) since 2000. These differences in richness represent a rate of increase of 1.6% per year for reptiles in general, 1.7% for squamates, and 1.9% per year for lizards.

**Table 1:** Summary of family, genera and species diversity of world’s reptiles.

Group	Number of Families	Number of Genera	Number of Species
**Reptiles**	**82**	**1,131**	**9,546**
**Turtles**	14	93	327
**Crocodilians**	3	9	25
**Tuataras**	1	1	1
**Squamata**	**64**	**1,028**	**9,193**
**Lizards**	35	498	5,634
**Snakes**	23	511	3,378
**Amphisbaenians**	6	19	181

For convenience, reptiles in general and Squamata (lizards, snakes and amphisbaenians) lineage richness are shown separately.

**Figure 2:**
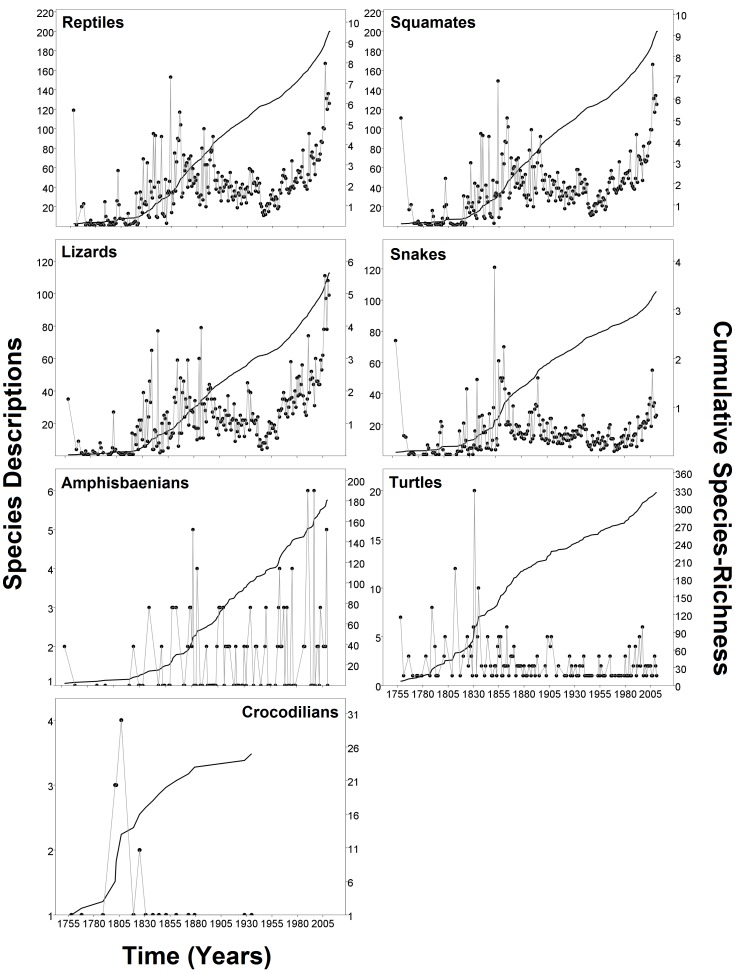
Historical rates of reptile species descriptions (dots) and rate of accumulation of new species (continuous line) since Linnaeus (1758), to the most recent species appeared to March 2012. The two top plots show rates for reptiles as a whole and for the squamate clade, respectively, while the remaining ones focus on major reptile groups. The tuatara is not shown given the single-species richness of the order Rhynchocephalia. The time scale shown in the bottom plots is identical to the timescales of the plots above them.

Historically, the rates of new species descriptions have been highly asymmetric among time periods, and among major reptile groups (Fig. 2). Given that most reptiles are squamates, the historical trends found in squamates and reptiles in general are almost identical. The description rates of crocodilians and turtles were considerably higher during the first half of the 19^th^ century, followed by conspicuous declines. Descriptions of lizards and snakes (and hence, of squamates together), on the other hand, have peaked in different historical periods. While three peaks standout in the history of snakes, two main periods of lizard descriptions are seen (with an additional early weak peak), as Linnaeus named many more snakes than lizards (Fig. 2). The description rates of new lizard species have increased dramatically during the 21^st^ century to an unprecedented level compared to any reptile group at this period. For snakes, the highest proportion of species was described during the 1850s and 1860s, although the numbers of descriptions have increased in the last two decades as well. The historical tendency for descriptions of amphisbaenians is clearly more similar to the historical rates of lizards (Fig. 2). Interestingly, a sharp decline in reptile species descriptions, especially in lizards and snakes, occurred between approximately the 1940s and the 1970s (Fig. 2). Overall, the last five years have seen the highest description rates of reptiles ever [31]. The year 2012 will surely enter the list, as, at the time of writing, >160 new species were already described (126 of which are lizards, the highest figure ever, and nearly all the rest are snakes).The cumulative curves of species-richness remain similar among all reptile groups, except for crocodilians, in which the curve has plateaued late in the 19^th^ century following a peak of species descriptions between 1800–1825, when more than half of all species were described (Fig. 2).

### Taxonomic Imbalance

**Figure 3:**
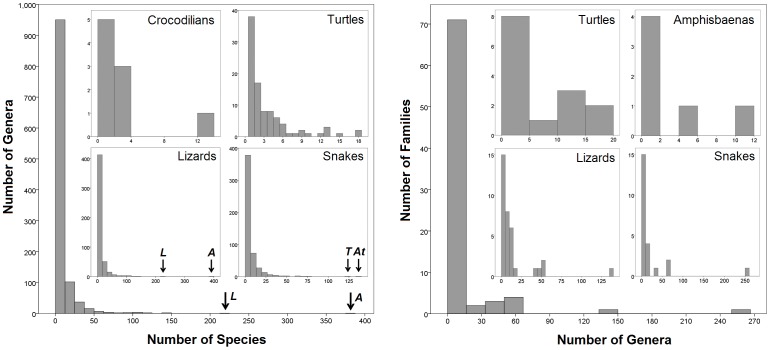
Frequency distributions of reptile biodiversity. The left plot depicts the overall frequency distribution of species per genera for all reptiles together, and the distributions for major clades separately (tuataras and amphisbaenians not shown) in the inset plots. For lizards and reptiles in general, the genera *Anolis* (*A*) and *Liolaemus* (*L*), and for snakes *Atractus* (*At*) and *Typhlops* (*T*) are indicated with black arrows. The right plots depict the same distributions, but for genera within families. Crocodilians and tuataras are not shown given the low number of families and genera.

**Figure 4:**
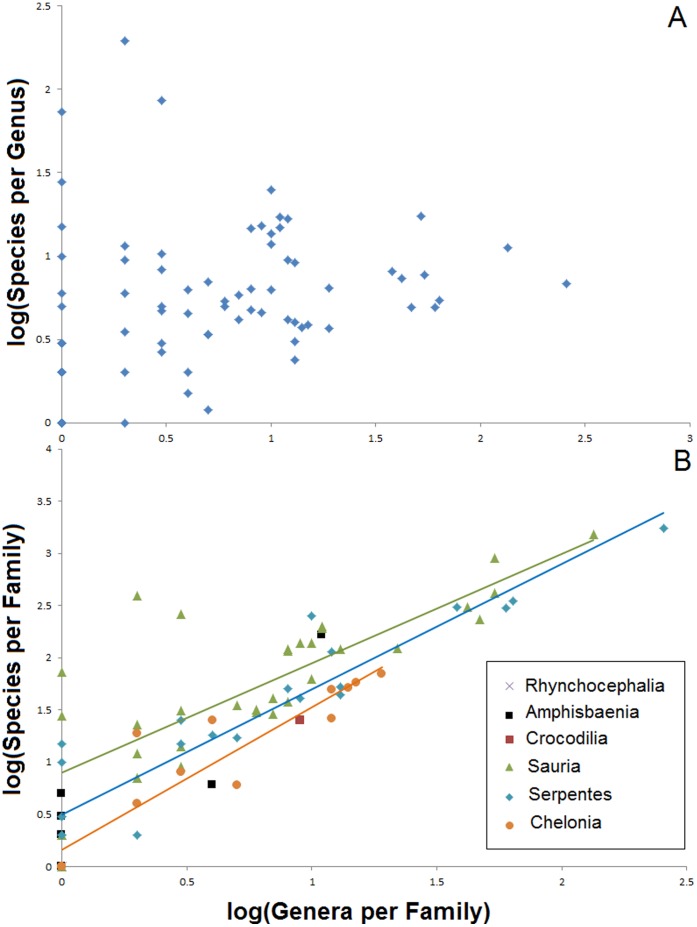
Species richness in reptilian taxa. Overall, the number of species per genus in a family is not directly correlated with the number of genera per family (A). However, the number of genera is proportional to the number of species per family in all major reptile groups (B). Each data point represents a family.

**Figure 5:**
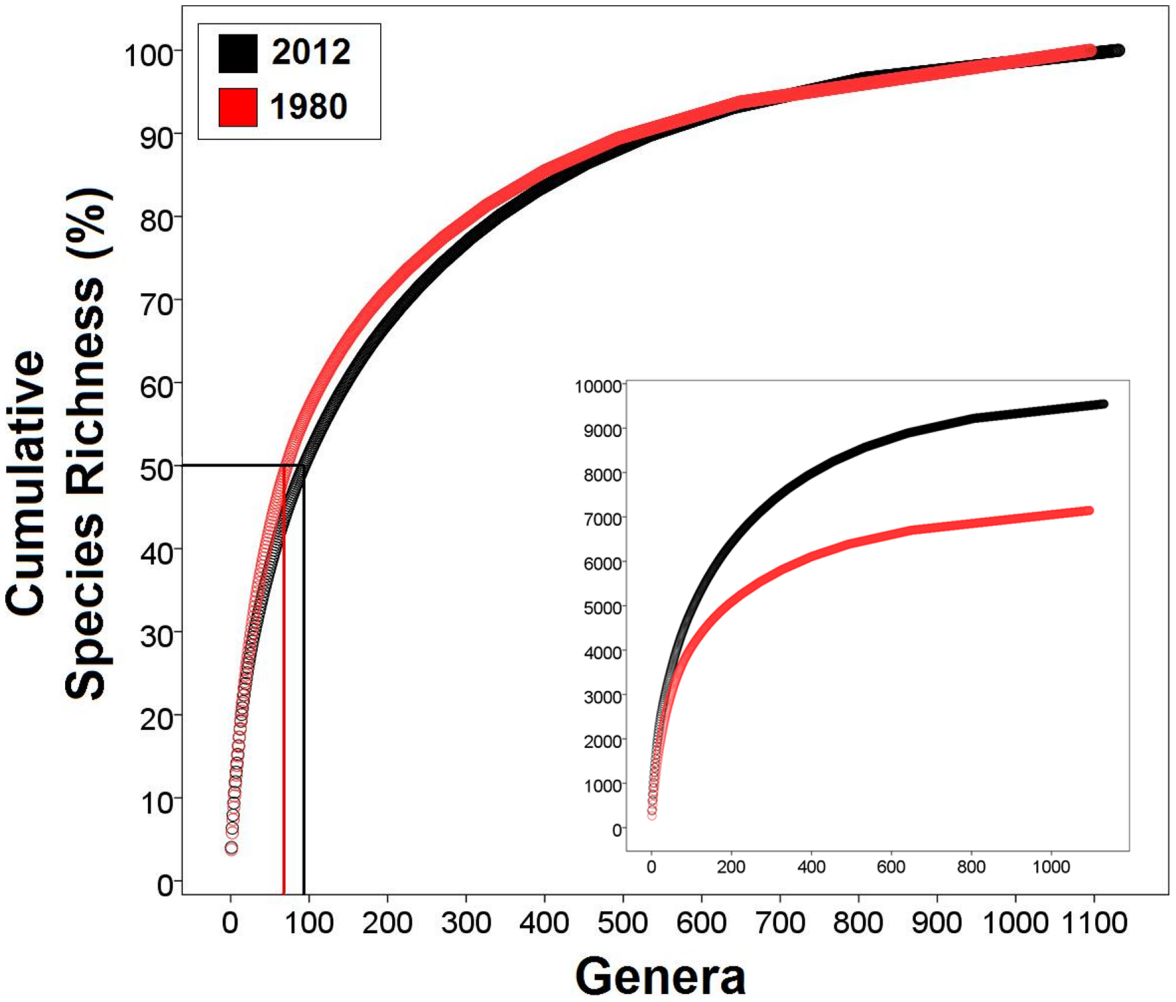
Distribution of the world’s reptile species as the accumulation of relative diversity from the richest (*Anolis*) to the poorest genera based on a 2012 and a 1980 dataset (main plot). The 2012 relationship reveals that 50% of global reptile diversity is accounted for by the 93 richest genera only, all of them squamates, and 92 being lizards and snakes. The inset plot displays the accumulation of species for both datasets as absolute species numbers per genera. *Anolis* is treated as a single large genus (see text for details).

Analyses of the frequency distributions of reptile richness within major taxa consistently reveal strong, significant, right-skewed distributions (genera within families: Skewness = 5.5, SE = 0.27, test (Shapiro-Wilks) = 0.38 (82 *df*), *P*<0.0001; species within genera: Skewness = 4.5, SE = 0.27, test = 0.43 (82* df*), *P*<0.0001; species within genera: Skewness = 9.7, SE = 0.07, test = 0.35 (1131 *df*), *P*<0.0001; tests for reptile orders reveal qualitatively identical results). Thus, most families and genera consist of few genera and species, respectively, while very rich lineages are rare (Fig. 3). This distribution of diversity remains constant for all reptiles together, for different reptilian taxa separately, and when these analyses are conducted both for numbers of species within genera and for the numbers of genera within families (Fig. 3). Therefore, this organization of reptile diversity is not affected by taxon (species, genera) richness. In addition, the number of species per genus in a family is not predicted by the number of genera per family (Fig. 4A). However, the number of genera is positively correlated with the number of species per family in all major reptile groups (Lizards: *R* ^2^ = 0.61, *F*_1,32_ = 50.82, *P*<0.0001; snakes: *R* ^2^ = 0.87, *F*_1,21_ = 137.5, *P*<0.0001; amphisbaenians: *R* ^2^ = 0.81, *F*_1,4_ = 16.77, *P* = 0.01; turtles: *R* ^2^ = 0.86, *F*_1,12_ = 76.1, *P*<0.0001; Fig. 4B). In line with these observations, a further analysis shows that 50% of the world’s reptile species diversity is accounted for by only 93 genera (8.2% of all reptile genera, all of them squamates), whereas the remaining 50% of the species are spread across the other 1,038 genera (Fig. 5). Indeed, the ten richest reptile genera (0.9% of the total 1,131 genera) contain 1,553 species in total, which represents 16.3% of global reptile biodiversity.

## Discussion

### The Distribution of Richness in Reptile Taxa

Reptiles, with 9,546 species (and ∼2,800 subspecies), are the second richest class of tetrapods – close to the ∼10,600 known species (and ∼12,000 subspecies) of birds [37], and substantially more diverse than the ∼6,770 species of amphibians [38], and the ∼5,400 species of mammals [39], [40]. Most reptile diversity is concentrated in the hyper-diverse clade Squamata (Fig. 1), within which a 98% of the diversity is concentrated in lizards (a paraphyletic grouping) and snakes. Therefore, the high diversity of Squamata is mostly responsible for the prominent global biodiversity of reptiles as a whole.

Our observations reveal that historical rates of newly described species for the three squamate groups separately are clearly similar (multimodal), while these rates differ importantly from those found in turtles and crocodilians (Fig. 2). Hence, the intrinsic species-richness of clades is not a consistent predictor of description rates as turtles are more species rich than amphisbaenians, yet this latter clade of squamates exhibits similar rates to the other two, extremely rich, squamate groups (Fig. 2). Indeed, it is interesting to note that the rates of species descriptions of amphisbaenians have increased importantly over the last three decades. This suggests that a more intense search for secretive species coupled with modern techniques for taxonomic inference (e.g., molecular systematics) may reveal new species of amphisbaenians that may have remained unappreciated. Overall, it can be speculated that the large range-size and large body size of turtles and crocodilians may have resulted in rapid and early discovery and description of most species, while new lizard and snake species (and, potentially, amphisbaenians) continue to be reported at high rates given their high intrinsic diversity (except for amphisbaenians), which seems to be related to small body size and small geographic ranges (see e.g., [29], [41], [42]). On the other hand, the historical tendencies of the accumulation of species richness are remarkably similar among all groups except crocodilians, in which the curve has plateaued after an active period of species descriptions early in the 19^th^ century (Fig. 2).

### The Nature of Reptile Biodiversity

The analyses of lineage diversity conducted on our global dataset reveals a qualitatively similar and strong tendency for right skewed frequency distributions of lineage richness, where most groups consist of a few lineages (Fig. 3). Interestingly, these richness distributions are consistent both among major clades and across different hierarchical levels in the reptile phylogeny. Thus, our results show that reptiles in general, and major groups within reptiles separately, mostly contain genera with only few species, and most families have few genera (see also [25]). This means that extremely diverse lineages are rare, yet, represent major contributions to the total diversity of the group [25], [32]. In fact, as shown earlier in this paper, the ten richest reptile genera contain 1,553 species in total (16.3% of global reptile diversity; see also Fig. 5). Therefore, the existence of this constant pattern of across-clade and across-taxonomic scale diversity is not only consistent with similar patterns observed in other organisms [32], [43], [44], but also supports the prediction that biodiversity in reptiles is fractal [32] (i.e., the organization of diversity is scale-invariant, and hence, remains similar at different taxonomic levels).

An important implication of these findings is that the total species richness of reptile families is caused by the disparate diversity of only a few genera. The rarity of exceptionally species-rich taxa suggests that a number of organismal and environmental conditions have to be met to initiate and maintain such high rates of evolutionary proliferation. Adaptive radiation theory posits that prominent radiations require both innovative traits (‘key innovations’) that allow the exploitation of resources in novel ways [19], [45], [46], and the existence of available resources to be exploited in the first place to consolidate a new niche for a newly forming species [45], [47]. When no niches are available, diversification rates are expected to decline as a result of density-dependent effects due to saturation of ecological opportunity [48]. Globally, only a few reptile lineages have met these conditions in unusually favourable combinations. Most notably, the two richest reptile genera, *Anolis* and *Liolaemus*, have evolutionarily outperformed all other reptile (and even amniote) genera in terms of species diversity (Table 2). It should be noted, however, that the split of *Anolis* into multiple genera has previously been suggested in multiple papers [16], [17], and hence, according to these views the family Dactyloidae consists of eight, rather than one, genera. Anoles, on the one hand, appear to have accessed a variety of novel niches by acquiring subdigital toepads that facilitated unprecedented exploitation of arboreal microhabitats, while reinforcing speciation rates via dewlap-based communication [11]. The *Liolaemus* radiation, on the other hand, is likely to have been promoted by the uplift of the Andes, which opened enormous ecological opportunities to be exploited [24], accompanied by the subsequent colonization of Patagonia (possibly facilitated by the Andean bridge itself). Indeed, the uplift of the Andes has increasingly been implicated in the proliferation of high biodiversity in other organisms [49]–[51]. The access to such ecological opportunities appears to have been facilitated by the adaptive potential of *Liolaemus* to exploit all possible structural and thermal microhabitats [15], [52]–[55], food resources [56], and to evolve alternative life history strategies to reproduce efficiently across extreme climatic gradients [57], [58]. As a result, *Liolaemus* species are the dominant (and in extreme elevations and latitudes, sometimes the only) reptiles in most areas of their distribution [14], [15], [59]–[61]. In *Liolaemus*, the identification of underlying key innovations remains less clear, although multiple independent episodes of evolution of viviparity have apparently opened multiple opportunities to colonize cold climates [24]. It remains unclear whether the ages of the *Anolis* and *Liolaemus* radiations are linked to their current differences in diversity, although the *Liolaemus* radiation seems to be considerably younger than *Anolis*. While estimates suggest that *Anolis* may have radiated for at least 60+ My [11], [16], *Liolaemus* is estimated to have radiated for 20+ My [62], [63]. Interestingly, the highly diverse gecko genus *Cyrtodactylus* has also been estimated to have originated about 60 My ago [64], which reinforces the idea that the *Liolaemus* radiation has been remarkably rapid. However, in general, these estimates have large margins of error and overlap to certain extent [11], which makes difficult to fully appreciate the temporal asymmetries behind the radiations of these lineages.

**Table 2:** The top ten richest genera of reptiles (to March 2012).

Genus	Species	Family	Type
***Anolis*** 1	384	Dactyloidae	Lizard
***Liolaemus***	223	Liolaemidae	Lizard
***Cyrtodactylus***	149	Gekkonidae	Lizard
***Atractus***	138	Colubridae	Snake
***Typhlops***	123	Typhlopidae	Snake
***Sphenomorphus***	122	Scincidae	Lizard
***Hemidactylus***	111	Gekkonidae	Lizard
***Cnemaspis***	103	Gekkonidae	Lizard
***Amphisbaena***	100	Amphisbaenidae	Amphisbaenian
***Ctenotus***	100	Scincidae	Lizard

1 sensu lato.

Note that the list contains squamates only, of which most are lizards, including the three largest genera. Of the 20 richest genera, 14 are lizards, five are snakes and one is an amphisbaenian.

The phylogeny of global reptiles is fast advancing with multiple recent studies enabling a deeper understanding of both the relationships among major clades [65], [66] and within species-rich lineages [64]. However, a well-resolved, dated, species-level reptile phylogeny remains unavailable. Several rich groups with high potential as model organisms, such as *Liolaemus* for example, require substantial further efforts to achieve even nearly complete phylogenies. As such reptile phylogenies become available, more comprehensive and sophisticated tests of central hypotheses on reptile diversification and extinctions (and thus their combined contribution to the evolution of biodiversity) will be possible, to ultimately strengthen conclusions on the mechanisms and processes underlying the history, present and future of these vertebrates.

### Discrete Linnaean Categories and Darwin’s Tree of Life

Modern evolutionary biology reconciles Linnaeus’s [33] taxonomic system with Darwin’s [67] evolutionary tree of life under the view that biodiversity proliferates through the split of ancestors into (at least largely) genetically isolated categories [68]–[70]. However, these two views of nature sometimes conflict [71], mostly because the conceptual basis of both ideas differs importantly as Linnaeus’s system was established before organisms were described as a phylogenetic continuum under Darwin’s theory of descent with modification. Therefore, the application of taxonomic categories necessarily relies on arbitrary decisions on where the boundaries of these groups are, even if dealing with monophyletic groups only. Such arbitrariness inevitably dictates the direction of results. Hence, taxonomic rearrangements can alter the current shape of lineage diversity distributions. In order to test for such uncertainties, we have used the Reptile Database to identify the 7,145 reptile species that had been described by 1980, a time when taxonomy was primarily based on morphological traits. The number of genera considered valid in 1980 was similar to today’s (Fig. 5), and although the total number of species was considerably different, the overall frequency distribution is fundamentally the same (Fig. 5). However, there has clearly been a trend towards splitting during the past three decades, not the least because many groups have been shown to be polyphyletic. For instance, most Palearctic green lizards were then considered members of the genus *Lacerta*, which has subsequently been split into multiple genera [72]. Similar taxonomic splits into multiple smaller genera have been suggested for the richest reptilian genera (e.g., [14], [17]), including the recent split of *Anolis* [16], as well as for many other reptilian taxa (e.g., [73]). Given the large influence of only a few unusually rich clades on the frequency distributions of taxonomic richness in reptiles (Fig. 3), which are at the same time the clades more likely to be split up, the arbitrariness of Linnaean taxonomic practice can often alter the perceptions of biodiversity if based on taxonomy. Clearly, these limitations will remain prevalent until a more objective, phylogenetic based system of organismal classification is generally employed.

## Materials and Methods

### Data Sources

Our study relies on a complete dataset covering the entire global diversity of living reptiles known to March 2012, which has been taken from the online Reptile Database [13]. The database is the repository of the data which we employed to identify lineage richness at different phylogenetic levels, from total reptile diversity to species richness per genus. We ignored subspecies, and hence, our reported results are entirely based on taxa with currently accepted full-species status.

### Phylogeny and Taxonomic Richness

The phylogenetic organization of the data was based on a composite family-level tree encompassing the entire class Reptilia, which we assembled from recent phylogenetic hypotheses presented for lizards in general [74], snakes [75], turtles [76], [77] and crocodilians [3], [78]. The phylogenetic relationships among these major groups have been reported in a number of other studies (e.g., [3], [79]–[82]). Among these phylogenetic-based taxonomic decisions, we follow Townsend et al.’s [83] recent proposition to separate the paraphyletic family Polychrotidae into Polychrotidae for the genus *Polychrus*, and Dactyloidae for the genus *Anolis* (see Fig. 1). However, given that the separation of the genus *Anolis* into eight different genera requires further validation, we comply to the traditional view that maintains these genera names under *Anolis* [11], [16]. These data were employed to conduct analyses of distribution of lineage (including species) richness within and among clades. A general picture of the richness distribution of diversity along the reptilian phylogeny is presented in Figure 1. We then plotted the frequency distributions of species within genera, and of genera within families, across all reptiles, and separately for each major group to show among-group contrasts, at different phylogenetic scales (e.g., Fig. 3).

### Species Descriptions

To reconstruct the historical patterns of frequency in new species descriptions (from 1758, which includes the first species named by Linnaeus, to 2012), we obtained the year of publication of all currently recognized reptile species. Therefore, names currently recognized as junior synonyms in the Reptile Database [13] have been ignored. We plotted historical trends of species descriptions for all reptiles, and then separately for each major reptile group (Fig. 2). These analyses substantially expand the general overview (for reptiles as a whole) previously presented by Uetz [31]. The species taxonomic diversity as of 1980 (used for Fig. 5) was compiled from the historical (or synonymy) records of the Reptile Database. The names used in 1980 or the most recently used names before 1980 were used as the 1980 names for genera. For instance, the genus *Rhinotyphlops* contained 22 species in 1980 while it contains only four species today after having been split up into multiple genera such as *Letheobia* and others. While the incompleteness of the synonymy most likely has caused some inconsistencies, the overall pattern of species richness of genera (Fig. 5) appears to be unaffected.
